# Supplementary material for: Comparison of the Opn-CreER and Ck19-CreER Drivers in Bile Ducts of Normal and Injured Mouse Livers
Source: Cells. 2019 Apr 25;8(4):380. doi: 10.3390/cells8040380 (PMC6523626; doi:10.3390/cells8040380)
Supplement: Supplementary file 1 [file cells-08-00380-s001.zip › FigS1.pdf]

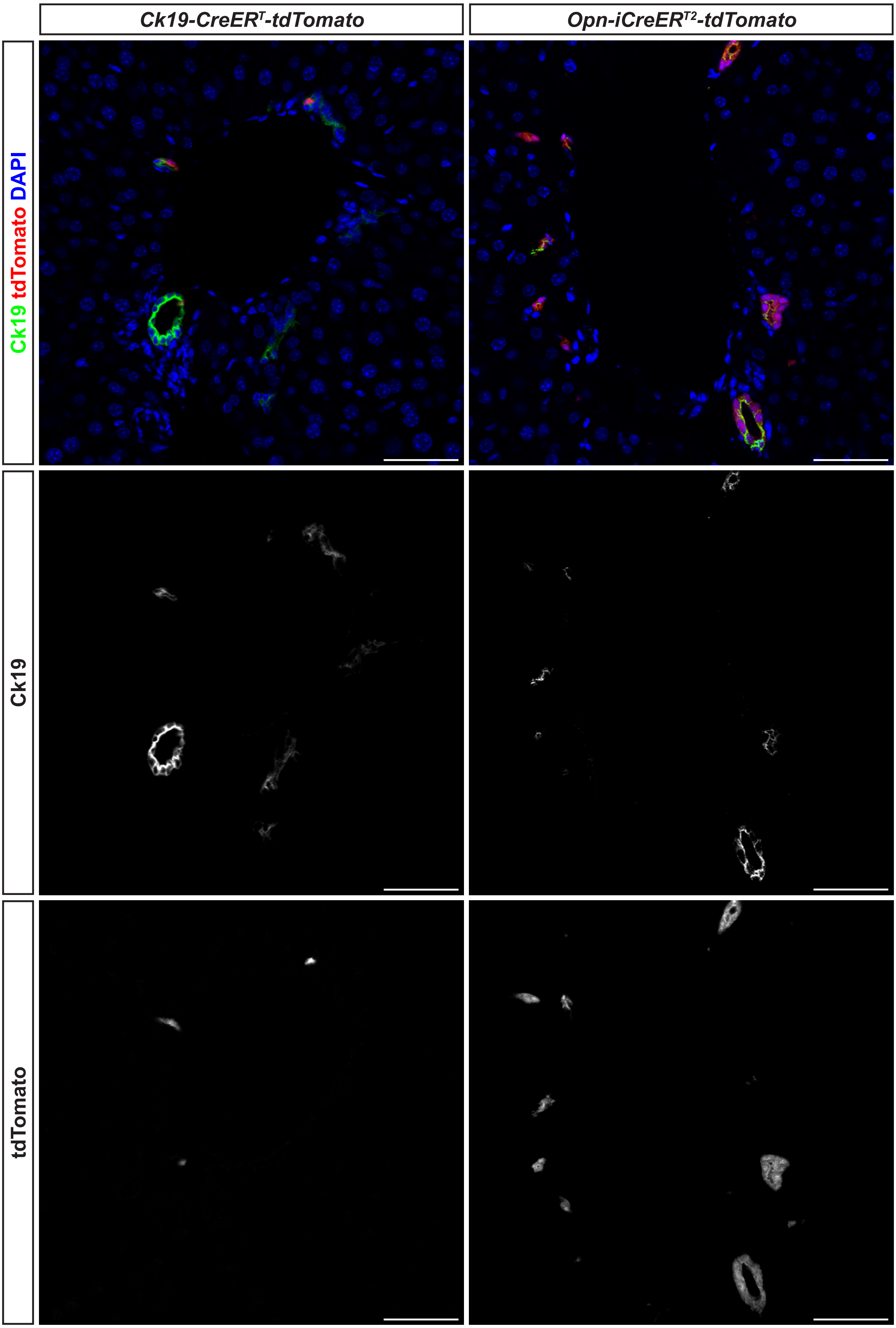

Figure S1. Troma III Ck19 antibody labels all cholangiocytes.
Immunofluorescent detection of Ck19 with tdTomato in *Ck19-CreER-tdTomato* and *Opn-CreER-tdTomato* mice. One portal region is shown. Scale bars: 50 μm.
