# Supplementary material for: Comparison of the Opn-CreER and Ck19-CreER Drivers in Bile Ducts of Normal and Injured Mouse Livers
Source: Cells. 2019 Apr 25;8(4):380. doi: 10.3390/cells8040380 (PMC6523626; doi:10.3390/cells8040380)
Supplement: Supplementary file 1 [file cells-08-00380-s001.zip › FigS2.pdf]

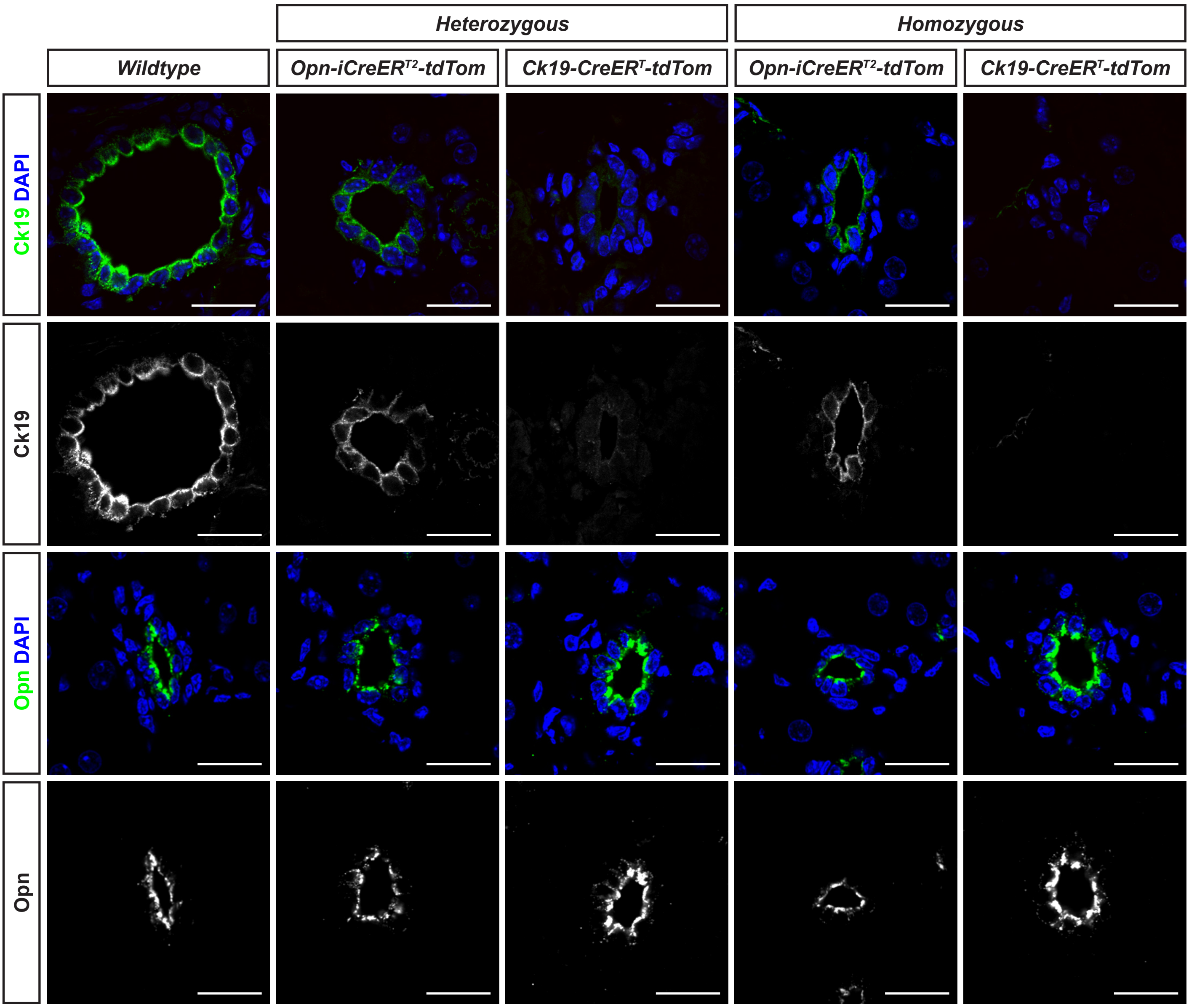

Figure S2. Ck19 expression is abrogated in homozygous *Ck19-CreER-tdTomato* mice. Immunofluorescent detection of Ck19 and Opn in heterozygous and homozygous *Ck19-CreER-td-Tomato* and *Opn-CreER-tdTomato* mice. Scale bars: 20 µm.
