# Supplementary material for: Comparison of the Opn-CreER and Ck19-CreER Drivers in Bile Ducts of Normal and Injured Mouse Livers
Source: Cells. 2019 Apr 25;8(4):380. doi: 10.3390/cells8040380 (PMC6523626; doi:10.3390/cells8040380)
Supplement: Supplementary file 1 [file cells-08-00380-s001.zip › FigS4.pdf]

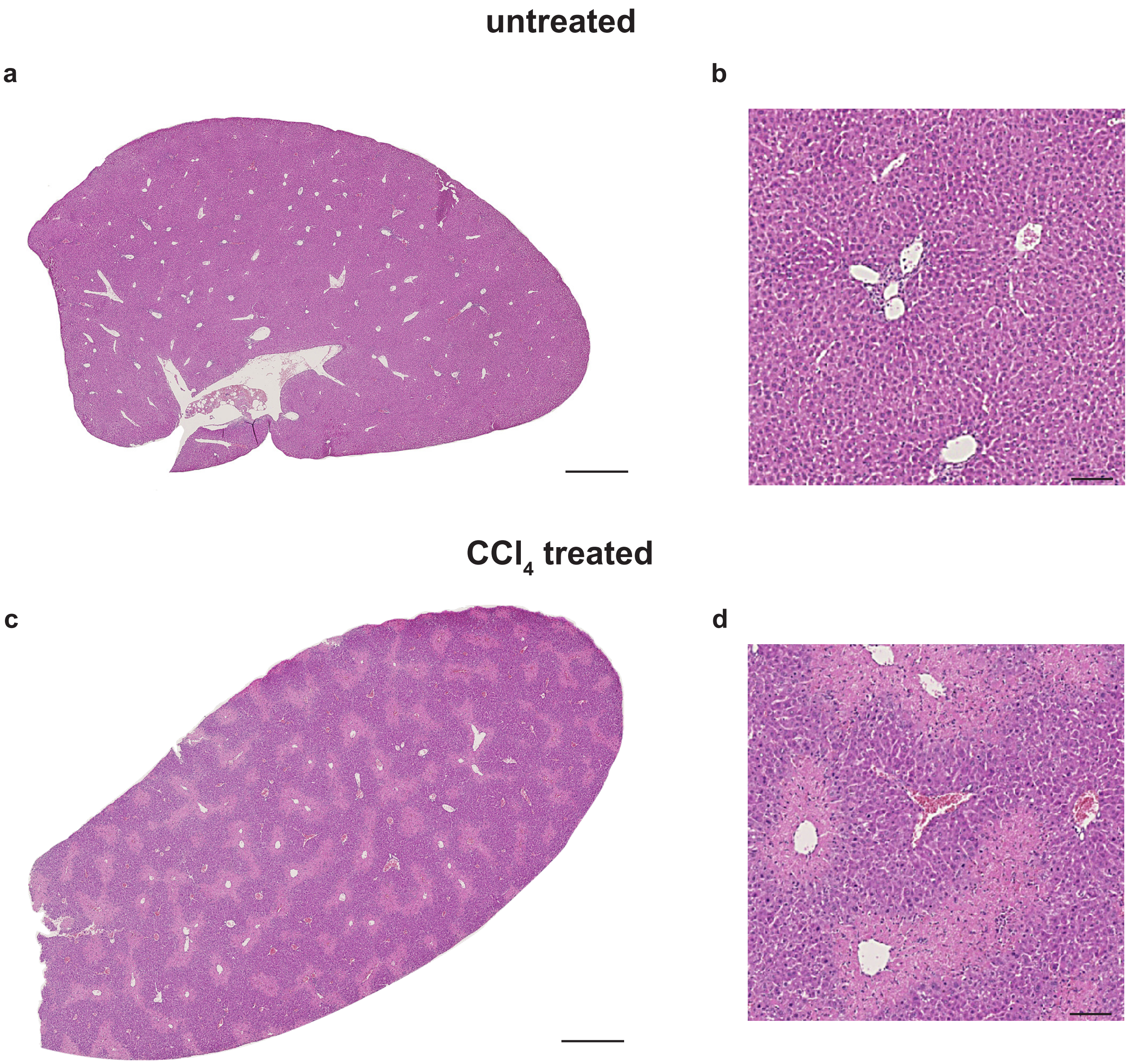

Figure S4. CCl_4 injection causes necrosis of centrilobular hepatocytes.
H&E staining on a section of a wildtype untreated liver (a,b) and 48h after CCl_4 injection (c,d). Scale bars (left): 1 mm, scale bars (right): 100 μm.
