# Supplementary material for: Comparison of the Opn-CreER and Ck19-CreER Drivers in Bile Ducts of Normal and Injured Mouse Livers
Source: Cells. 2019 Apr 25;8(4):380. doi: 10.3390/cells8040380 (PMC6523626; doi:10.3390/cells8040380)
Supplement: Supplementary file 1 [file cells-08-00380-s001.zip › FigS5.pdf]

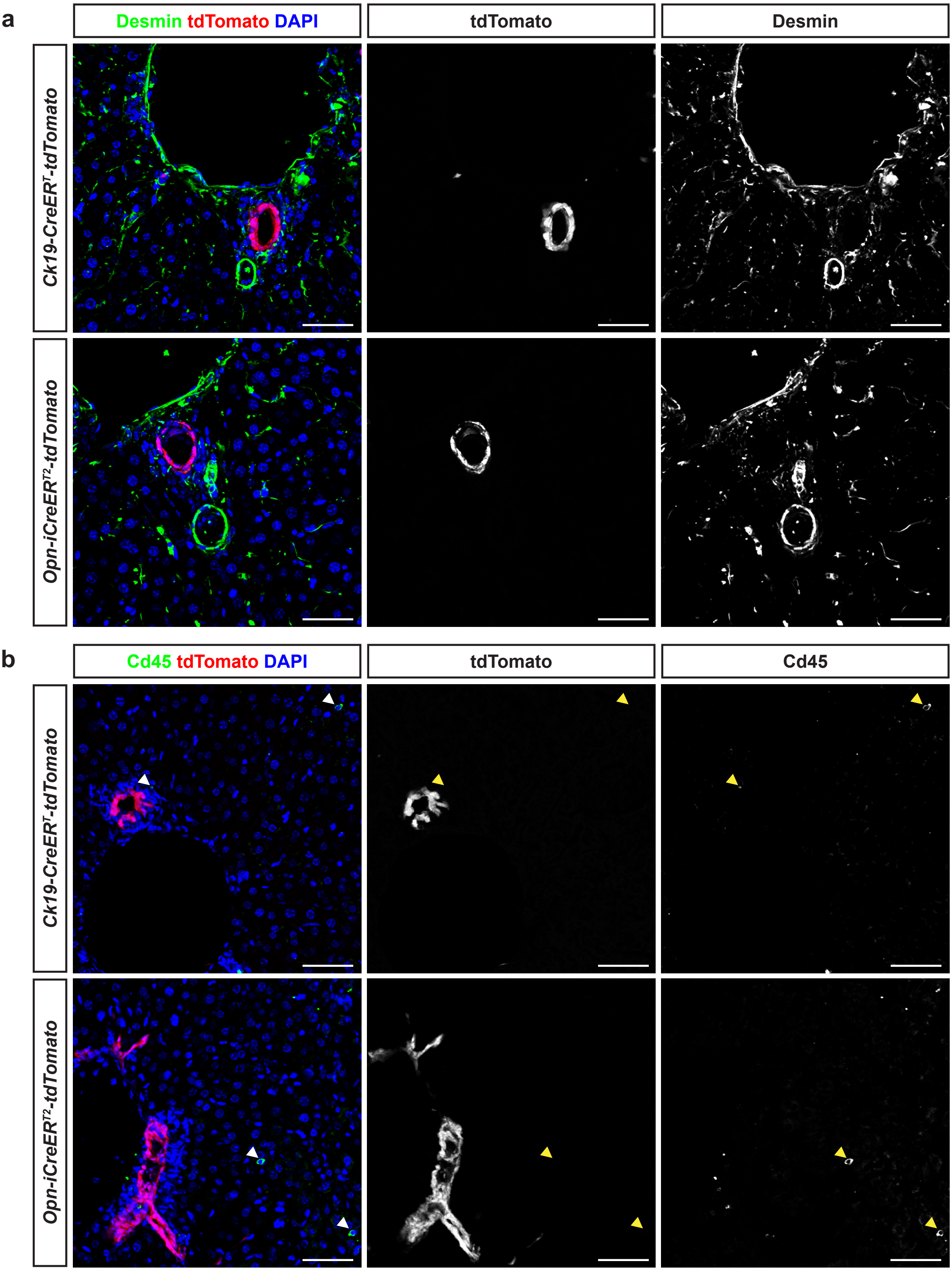

Figure S5. The Ck19-CreER^T and Opn-iCreER^{T2} drivers do not trigger recombination in hepatic stellate cells or immune cells after liver injury.
Immunofluorescent detection of Desmin (a) and Cd45 (b) with tdTomato on liver sections of *Ck19-CreER-tdTomato* and *Opn-CreER-tdTomato* mice three days after CCl_4 treatment. Arrow heads indicate Desmin+ and Cd45+ cells respectively. Scale bars: 50 µm.
